# Distinct gut microbial profile in PIT1 lineage PitNETs: a potential link to cognitive impairment

**DOI:** 10.1186/s41016-025-00421-7

**Published:** 2025-12-30

**Authors:** Junjun Li, Lingye Zhang, Chen Ma, Jiang Long, Jinpeng Lv, Xingli Deng

**Affiliations:** 1 Department of Neurosurgery, The First Affiliated Hospital of Kunming Medical University, Kunming, Yunnan 650032 China; 2 Department of Neurosurgery, Pu’er People’s Hospital, Yunnan, Pu’er 665000 China; 3 Department of Neurosurgery, Ningxia People’s Hospital, Yinchuan, Ningxia 750000 China

**Keywords:** Pituitary neuroendocrine tumors, Cognitive function, Montreal Cognitive Assessment, Hormone, Gut microbiota

## Abstract

**Background:**

Patients with pituitary neuroendocrine tumors (PitNETs) frequently experience cognitive impairment (CI), yet the underlying mechanisms remain poorly understood.

**Method:**

In this study, we assessed cognitive function in 42 PitNETs patients and 42 healthy controls using the Montreal Cognitive Assessment (MoCA), evaluating the effects of tumor volume, invasiveness, pituitary hormone levels, lineage, and surgical intervention.Furthermore, 16S rRNA amplicon sequencing of fecal samples was performed to reveal alterations in gut microbiota composition.

**Results:**

The results demonstrated significantly lower MoCA scores in PitNETs patients compared to controls. Patients with PIT1 lineage tumors exhibited more severe CI than those with SF-1 lineage tumors. Notably, surgical treatment led to improved cognitive performance. The sequencing revealed significant alterations in gut microbiota composition in PitNETs patients. Specifically, PIT1 lineage cases showed reduced levels of the butyrate-producing genus Agathobacter and increased abundance of UBA1819 and Alistipes indistinctus, taxa that have been implicated in pro-inflammatory states.

**Discussion:**

These preliminary findings suggest that PIT1-lineage PitNETs may be associated with an increased susceptibility to cognitive impairment, potentially involving interactions between hormonal dysregulation and gut microbiota dysbiosis. This exploratory hypothesis provides a conceptual framework for future research to elucidate underlying mechanisms and explore potential interventions for cognitive impairment in PitNETs.

**Supplementary Information:**

The online version contains supplementary material available at 10.1186/s41016-025-00421-7.

## Background

PitNETs are one of the most common primary central nervous system tumors, accounting for approximately 16% of all intracranial neoplasms [1], and have a reported prevalence of approximately 77.6 cases per 100,000 people. Imaging studies in the general population and autopsy samples from deceased individuals reveal a detection rate for PitNETs as high as 14.4% to 22.5% [2]. For patients with PitNETs, the primary clinical concerns typically involve mass effects caused by tumor compression of adjacent structures (e.g., visual impairment due to optic chiasm compression) or hormonal and metabolic disturbances resulting from endocrine dysfunction [3]. Nevertheless, less attention has been given to the alterations in brain morphology and cognitive function caused by PitNETs. Increasing research indicates that a significant portion of PitNETs patients experience neuropsychiatric disorders, with mild cognitive impairment (MCI) being the most prevalent.

In a comparative study of cognitive function between patients with Cushing’s disease and those with nonfunctional pituitary adenomas, it was found that 30% of the patients showed mild impairments in attention, processing speed, executive function, and visual memory [4]. A study on the relationship between cognitive dysfunction and white matter tracts in patients with prolactinomas found that compared with the healthy group, patients with prolactinomas had impaired short-term and long-term visual and verbal memory, attention, concentration, executive, and language functions [5]. Patients with Cushing’s disease and acromegaly have a higher propensity for emotional regulation and social cognitive disorders [6, 7]. These findings suggest that patients with PitNETs are susceptible to cognitive dysfunction. Clarifying the related factors causing mental disorders in PitNETs patients and conducting early detection and timely intervention are crucially important for preventing the occurrence of neuropsychiatric disorders in PitNETs patients.

At present, the factors causing neuropsychiatric disorders in patients with PitNETs are not completely clear. Some studies contend that it is related to tumor size. For example, Savastano L. E. et al. assert that large pituitary tumors with suprasellar posterior extension and craniopharyngiomas in the third ventricle can induce severe Korsakoff-like amnestic syndrome [8]. There are also studies that attribute it to the abnormal hormone levels resulting from the tumors. For instance, Algahtany et al. found a significant correlation between changes in growth hormone (GH) levels and variations in depressive symptoms among patients with GH adenomas [9]. Nevertheless, some researchers hold disparate viewpoints. Korali Z. et al. maintain that the type, size, hormone levels, and treatment modalities of PitNETs have no obvious correlation with the occurrence of neuropsychiatric disorders in PitNETs patients [10]. In addition, as an endocrine tumor, PitNETs can cause endocrine and metabolic disorders. Research has found that gut microbiota can influence brain function through neural, endocrine, immune, and metabolic pathways [11]. Changes in the gut microbiota can affect cognitive function and play an important role in the development of various neuropsychiatric diseases. Therefore, gut microbiota may play a role in the neuropsychiatric disorders of patients with PitNETs. In summary, the major factors influencing the cognitive function of patients with PitNETs are still not completely clear and thus call for further exploration.

To identify factors influencing cognitive function in patients with PitNETs, we conducted a prospective cross-sectional study involving 42 patients with PitNETs and 42 matched healthy controls. The study examined potential correlations between cognitive function (assessed by MoCA scores) and multiple variables, including tumor volume, invasiveness status, surgical history, and gut microbiota characteristics, to comprehensively explore determinants of cognitive performance in this patient population.

## Methods

### Study subjects

This study recruited PitNETs patients who were hospitalized in the Department of Neurosurgery, the First Affiliated Hospital of Kunming Medical University from April 2022 to October 2023, and selected a corresponding family member with similar age, education level, diet, and living environment to the enrolled patients as a healthy control. This study was performed in accordance with the Declaration of Helsinki and approved by the Ethics Committee of the First Affiliated Hospital of Kunming Medical University and obtained written informed consent from each subject.

#### Inclusion criteria for patients

- Age 18–60 years
- Received surgical operation via endoscopic endonasal transsphenoidal approach
- Diagnosed as PitNETs by pathological examination
- Received surgical treatment for the first time and did not receive any treatment (including medication treatment and radiotherapy) before surgery
- No history of psychotropic drug use and no antibiotic treatment in the past 3 months

#### Exclusion criteria of patients

- With severe cardiovascular and cerebrovascular comorbidities (such as cerebral hemorrhage, cerebral infarction, and myocardial infarction)
- With significant mental health disorders
- Field-of-view loss more than 75%
- Deafness or aphasia
- Failed to complete primary education or education years (education years refer to the total number of years an individual has spent in formal education, from primary school through higher education) less than 6 years
- Serious complications after surgery (such as cerebrospinal fluid leakage, central nervous system infection, hydrocephalus, and epilepsy)

#### Inclusion criteria of control subjects

- No history of serious cardiovascular disease, cerebrovascular disease, or neuropsychiatric disease
- No history of psychotropic drug use and no antibiotic treatment in the past 3 months
- Completion of primary education or education years more than 6 years

After patient admission, basic information such as age, gender, education, body mass index (BMI), tumor volume, and history of hypertension and diabetes was collected. Blood lipids (including total cholesterol, triglycerides, high-density lipoprotein, and low-density lipoprotein), thyroid function, and pituitary-related hormones (including GH, insulin-like growth factor-1 (IGF-1), follicle-stimulating hormone (FSH), luteinizing hormone (LH), prolactin (PRL), thyroid-stimulating hormone (TSH), cortisol, and adrenocorticotropic hormone (ACTH) were detected. Patients with abnormally elevated hormone levels were classified as functional PitNETs (F-PitNETs) patients, while those with normal hormone levels were classified as nonfunctional PitNETs (NF-PitNETs) patients. Fecal samples were collected from all participants prior to surgery. Crucially, sample collection was performed before the initiation of any pharmacological treatment; therefore, none of the patients had been exposed to steroids, proton-pump inhibitors (PPIs), or dopamine agonists at the time of sampling. The samples were immediately frozen using liquid nitrogen and subsequently stored at − 80 °C until further processing.

### Tumor volume and invasiveness assessment

**Fig. 1:**
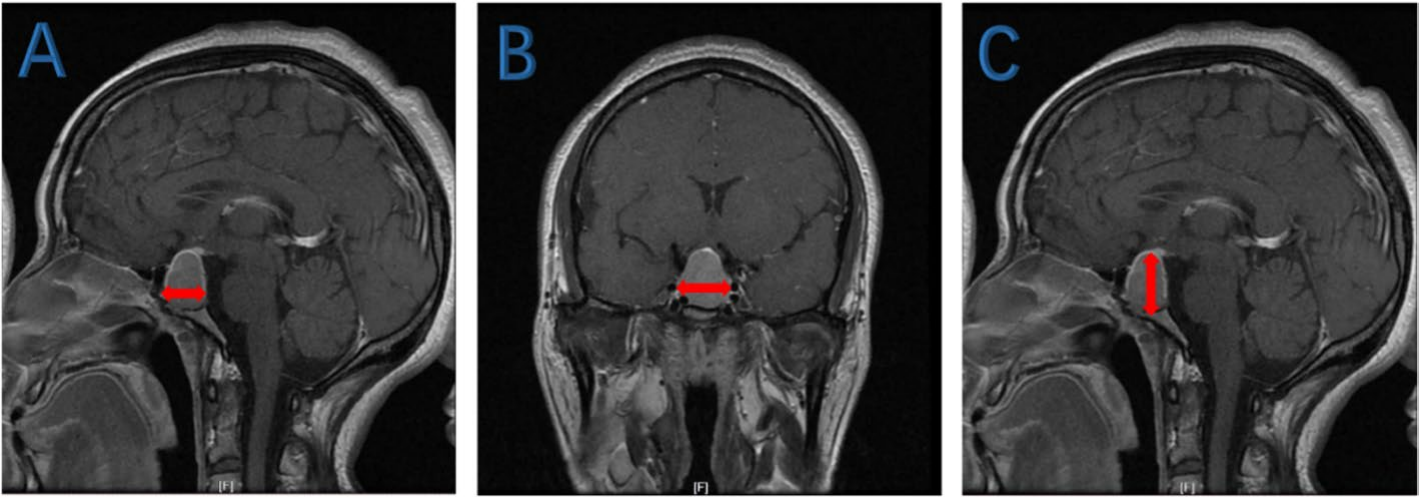
Tumor volume evaluation. **A** The maximum anterior–posterior diameter. **B** The maximum left–right diameter. **C** The maximum upper–lower diameter

All patients underwent MRI scanning of the pituitary gland before surgical treatment. Tumor size was evaluated based on the pituitary MRI assuming that the tumor was ellipsoidal. The maximum diameters of the tumor in three dimensions (anterior–posterior diameter, left–right diameter, and upper–lower diameters as shown in Fig. 1) were measured. Tumor volume was calculated using the formula V = 4πabc/3, where a, b, and c represent the maximum diameters along the three orthogonal dimensions.

Pituitary MRI imaging was used to assess tumor invasiveness, utilizing the modified Knosp grading system to characterize tumor invasion into the cavernous sinus, with grades 0 to 4 scoring 1, 2, 3, 4, and 5 points, respectively. The Hardy–Wilson score was used to evaluate the extent of extrasellar extension and sellae destruction, with stages A to E scoring 1, 2, 3, 4, and 5 points, respectively. Tumors with a single-item score ≥ 4 points or the sum of two scores ≥ 6 points were classified as invasive PitNETs (I-PitNETs), while others are categorized as noninvasive PitNETs (NI-PitNETs).

### Cognitive function assessment

MoCA was used to measure cognitive function 1–2 days before surgery and 3 months after surgery, and cognitive scores were calculated. The evaluation was conducted by the same team of physicians in the same location, in a quiet and comfortable environment. The test was completed by the subjects individually, without any accompaniment, and the entire testing process lasted approximately 30 min.

### Analysis of gut microbiota characteristics

The characteristics of gut microbiota were analyzed by 16S rRNA amplicon sequencing of fecal samples. Total DNA was extracted from fecal samples using the cetyltrimethylammonium bromide method. Throughout the DNA extraction process, negative controls (extraction blanks) without any sample were included to monitor potential contamination present in the extraction reagents. PCR amplification of the V3–V4 region of the 16S rRNA gene was performed using primers 341 F (5′-CCTAYGGGRBGCASCAG-3′) and 806R (5′-GGACTACNNGGGTATCTAAT-3′). During the PCR amplification step, no-template negative controls (PCR blanks) were processed simultaneously to identify any contamination introduced during the amplification process. The PCR products were purified using the Qiagen Gel Extraction Kit (Qiagen, Germany). Sequencing libraries were constructed using the TruSeq® DNA PCR-Free Sample Preparation Kit (Illumina, USA) and indexed. The quality of the libraries was assessed using the Qubit@2.0 Fluorometer (Thermo Scientific, USA) and the Agilent Bioanalyzer 2100 system (Agilent, USA). Finally, sequencing was performed on the Illumina NovaSeq 6000 platform (Illumina, USA) to obtain 250-bp paired end reads.

The data was processed according to the following steps: Demultiplexing of raw sequencing data was performed based on barcode sequences and PCR amplification primers to assign reads to corresponding samples. Paired-end reads were merged using FLASH (v1.2.7), and barcode/primer sequences were trimmed to generate raw tags. Quality filtering was applied to the raw tags using fastp (v0.23.1) to obtain high-quality Clean Tags.

To remove potential host contamination, all quality-filtered reads were aligned against the human reference genome (GRCh38) using Bowtie2 (v2.4.5). On average, 2.8% of sequencing reads per sample aligned to the GRCh38 and were removed as potential host-derived contamination. Chimeric sequences were detected and removed from the Clean Tags by alignment against the Silva database, resulting in Effective Tags. The Effective Tags were denoised using the DADA2 algorithm within QIIME2 (v2022.6) to derive the final amplicon sequence variants (ASVs) and feature table. Taxonomic annotation of ASVs was performed in QIIME2 using the Silva 138.1 database. A phylogenetic tree was constructed from all ASV sequences through multiple sequence alignment. Biodiversity abundance tables from kingdom to species level were generated based on annotated ASVs and sample metadata. Subsequent analyses included taxonomic composition profiling, differential abundance analysis, and cluster analysis across samples or groups.

All laboratory procedures, including DNA extraction, PCR amplification, and library preparation, were performed in a single batch under consistent conditions to minimize technical variability.

### Data analysis

All data were analyzed using SPSS 25 (IBM, Armonk, New York, USA). Quantitative data were expressed as mean ± standard deviation (x ± SD), and the differences between groups were analyzed by *T*-test. Qualitative data were analyzed by *χ*^2^ test. Paired sample *T*-test was used to analyze the difference in cognitive function before and after surgery. The variations in the distribution of gut microbiota among groups were analyzed using the nonmetric multidimensional scaling (NMDS) approach. The LDA effect size (LEfSe) analysis was employed to clarify the differences in the abundance of gut microbiota among each group. The bivariate correlation analysis was conducted by adopting the Spearman correlation analysis. The Benjamini–Hochberg procedure was applied to control the false discovery rate (FDR) for all hypotheses tested in the differential abundance analysis. Statistical significance was defined as *p* < 0.05.

## Results

### Demographic characteristics

We conducted a comparative analysis of age, sex, education, BMI, history of hypertension and diabetes, and blood lipids among PitNETs patients and healthy controls, I-PitNETs and NI-PitNETs patients, F-PitNETs and NF-PitNETs, and PIT1 and SF-1 lineage PitNETs patients. The results showed that there were no significant differences among the groups in these basic indicators (Table 1).

**Table 1:** Demographic characteristics of the subjects

Characteristic (*n*)	Sex (M/F)	Age (years)	Education (years)	BMI	Hypertension (Y/N)	Diabetes (Y/N)	TC (mmol/L)	TG (mmol/L)	HDL (mmol/L)	LDL (mmol/L)
Patient (*n* = 42)	23/19	42.81 ± 1.61	12.00 ± 0.52	24.87 ± 0.31	5/37	6/36	4.52 ± 0.17	2.28 ± 0.26	1.06 ± 0.05	2.65 ± 0.12
Control (*n* = 42)	16/26	39.93 ± 1.34	12.19 ± 0.47	24.21 ± 0.34	3/39	2/40	4.47 ± 0.14	2.45 ± 0.20	1.10 ± 0.05	2.59 ± 0.10
*P*-value	0.129	0.173	0.785	0.154	0.463	0.14	0.713	0.57	0.857	0.898
I-PitNETs (*n* = 22)	14/8	40.27 ± 2.21	12.50 ± 0.72	24.77 ± 0.41	2/20	4/18	4.34 ± 0.27	1.92 ± 0.29	1.08 ± 0.06	2.48 ± 0.15
NI-PitNETs (*n* = 20)	9/11	45.60 ± 2.24	11.45 ± 0.74	24.98 ± 0.66	3/17	2/18	4.73 ± 0.24	2.68 ± 0.47	1.03 ± 0.07	2.85 ± 0.21
*P*-value	0.138	0.099	0.315	0.784	0.566	0.461	0.283	0.171	0.653	0.161
F-PitNETs (*n* = 22)	11/11	40.50 ± 2.22	11.73 ± 0.69	25.47 ± 0.46	3/19	5/17	4.65 ± 0.27	1.88 ± 0.29	1.10 ± 0.05	2.50 ± 0.15
NF-PitNETs (*n* = 20)	12/8	45.35 ± 2.26	12.30 ± 0.78	24.21 ± 0.58	3/17	2/18	4.38 ± 0.22	2.72 ± 0.67	1.10 ± 0.72	2.83 ± 0.22
*P*-value	0.753	0.134	0.550	0.10	0.903	0.151	0.172	0.065	0.308	0.199
PIT1 lineage (*n* = 24)	11/13	40.25 ± 2.35	11.50 ± 0.68	25.44 ± 0.44	3/21	5/19	4.71 ± 0.28	2.57 ± 0.40	1.04 ± 0.05	2.73 ± 0.21
SF-1 lineage (*n* = 18)	12/6	46.22 ± 1.85	12.67 ± 0.79	24.11 ± 0.63	2/16	1/17	4.27 ± 0.19	1.89 ± 0.34	1.08 ± 0.81	2.55 ± 0.14
*P*-value	0.189	0.066	0.268	0.081	0.894	0.169	0.237	0.232	0.71	0.525

*BMI* body mass index, *TC* total cholesterol, *TG* triglyceride, *HDL* high-density lipoprotein, *LDL* low-density lipoprotein

### Cognitive function decline in patients with PitNETs

**Fig. 2:**
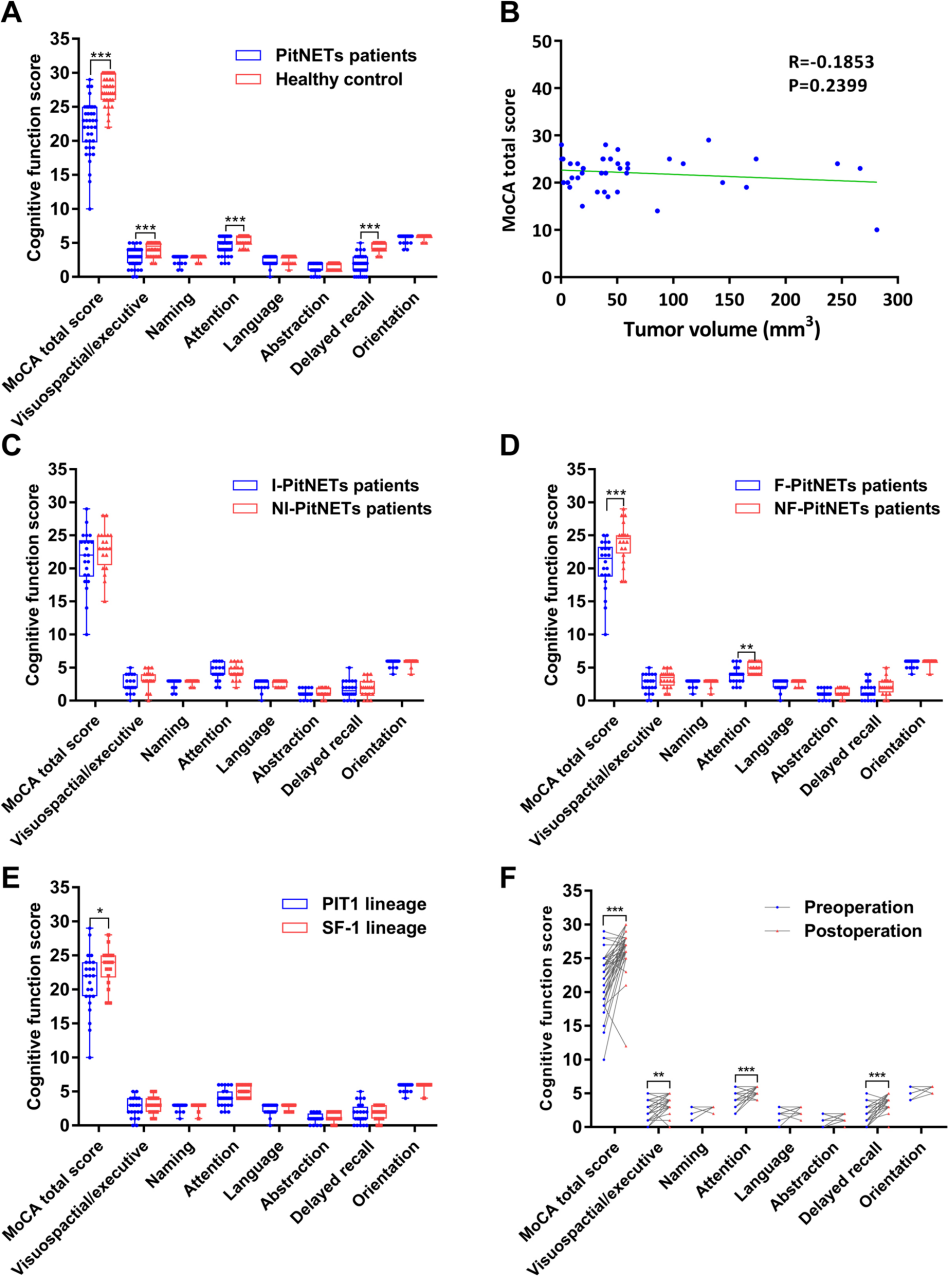
Cognitive function scores in different groups. **A** Cognitive function scores of PitNETs patients before surgery and healthy controls, ***P < 0.001. **B** Correlation analysis between tumor volume and cognitive function score, *R* = − 0.1853, *P* = 0.2399. **C** Cognitive function scores in patients with I-PitNETs and NI-PitNETs, *P* > 0.05. **D** Cognitive function scores in patients with F-PitNETs and NF-PitNETs, **P < 0.01, ***P < 0.001. **E** Cognitive function scores in PitNETs with PIT-1 and SF-1 lineage, *P < 0.05. **F** Cognitive function scores of PitNETs patients preoperative and postoperative, **P < 0.01, ***P < 0.001

Preoperative cognitive function test results showed that the cognitive function of PitNETs patients significantly declined compared with the control group. Specifically, according to the MoCA scale, PitNETs patients exhibited significant declines in visuospatial/executive function, attention, and delayed recall (Fig. 2A).

### Effect of tumor volume and invasiveness on cognitive function in patients with PitNETs

We measured and calculated the tumor volume and performed a correlation analysis between the tumor volume and the cognitive function score. The results showed that there was no significant correlation between the tumor volume and the cognitive function (*R* = − 0.1853, *P* = 0.2399) (Fig. 2B). Based on the modified Knosp grading system and the Hardy–Wilson scale, patients were divided into I-PitNETs and NI-PitNETs groups. Cognitive function test results showed no statistically significant difference in cognitive function between the two groups (Fig. 2C).

### Effects of pituitary hormone levels on cognitive function in PitNETs patients

According to the results of preoperative endocrine examination, the PitNETs patients were divided into two groups: F-PitNETs and NF-PitNETs groups. As shown in Fig. 2D, F-PitNETs patients had significantly lower cognitive function scores than NF-PitNETs patients (Fig. 2D).

### Differences in cognitive function among patients with different lineages of PitNETs

PitNETs are classified into three molecular lineages based on transcription factor expression: PIT1, SF-1, and TPIT. However, due to the low incidence of TPIT lineage tumors, the number of TPIT cases during our study period failed to meet the inclusion criteria. Consequently, this investigation focused exclusively on PIT1 and SF-1 lineage tumors. The results showed that the MoCA scores of the SF-1 lineage group were significantly higher than those of the PIT1 lineage group (Fig. 2E).

### Effect of transnasal endoscopic surgery on cognitive function in PitNETs patients

Cognitive function was reassessed for 3 months postoperatively in 42 patients with PitNETs, and the results were compared with their preoperative cognitive function scores. The results showed significant improvement in postoperative cognitive function scores compared with preoperative scores (Fig. 2F).

Comprehensive analysis of pituitary hormone levels before and 3 months after surgical intervention revealed distinct patterns of hormonal changes in patients with PitNETs. Quantitative comparisons demonstrated significant reductions in GH, IGF-1, and PRL levels following tumor resection. In contrast, no statistically significant differences were observed in FSH, LH, TSH, cortisol, or ACTH levels between preoperative and postoperative measurements (Table 2).

**Table 2:** Perioperative pituitary hormone levels in patients with PitNETs

Laboratory test items	Preoperation	Three months after the operation	*p*-value	*t*	*df*
GH (ng/ml)	6.605 ± 2.222, *n* = 42	0.9036 ± 0.195, *n* = 42	0.0124	2.556	82
IGF-1 (ng/ml)	562.3 ± 20.76, *n* = 15	290.3 ± 30.02, *n* = 15	< 0.0001	7.453	28
PRL(ng/ml)	122 ± 35.98, *n* = 42	34.92 ± 11.4, *n* = 42	0.0236	2.307	82
FSH (mIU/ml)	10.89 ± 2.612, *n* = 42	6.896 ± 1.098, *n* = 42	0.1628	1.408	82
LH (mIU/ml)	3.122 ± 0.5903, *n* = 42	3.191 ± 0.4554, *n* = 42	0.9269	0.0920	82
TSH (mIU/l)	2.472 ± 0.332, *n* = 42	1.811 ± 0.2151, *n* = 42	0.0989	1.669	82
8 am cortisol (pg/ml)	131.7 ± 9.258, *n* = 42	137.3 ± 11.25, *n* = 42	0.6995	0.3874	82
8 am ACTH (ng/ml)	15.51 ± 1.316, *n* = 42	15.17 ± 1.425, *n* = 42	0.8642	0.1716	82

### Analysis of gut microbiota characteristics in patients with PitNETs

**Fig. 3:**
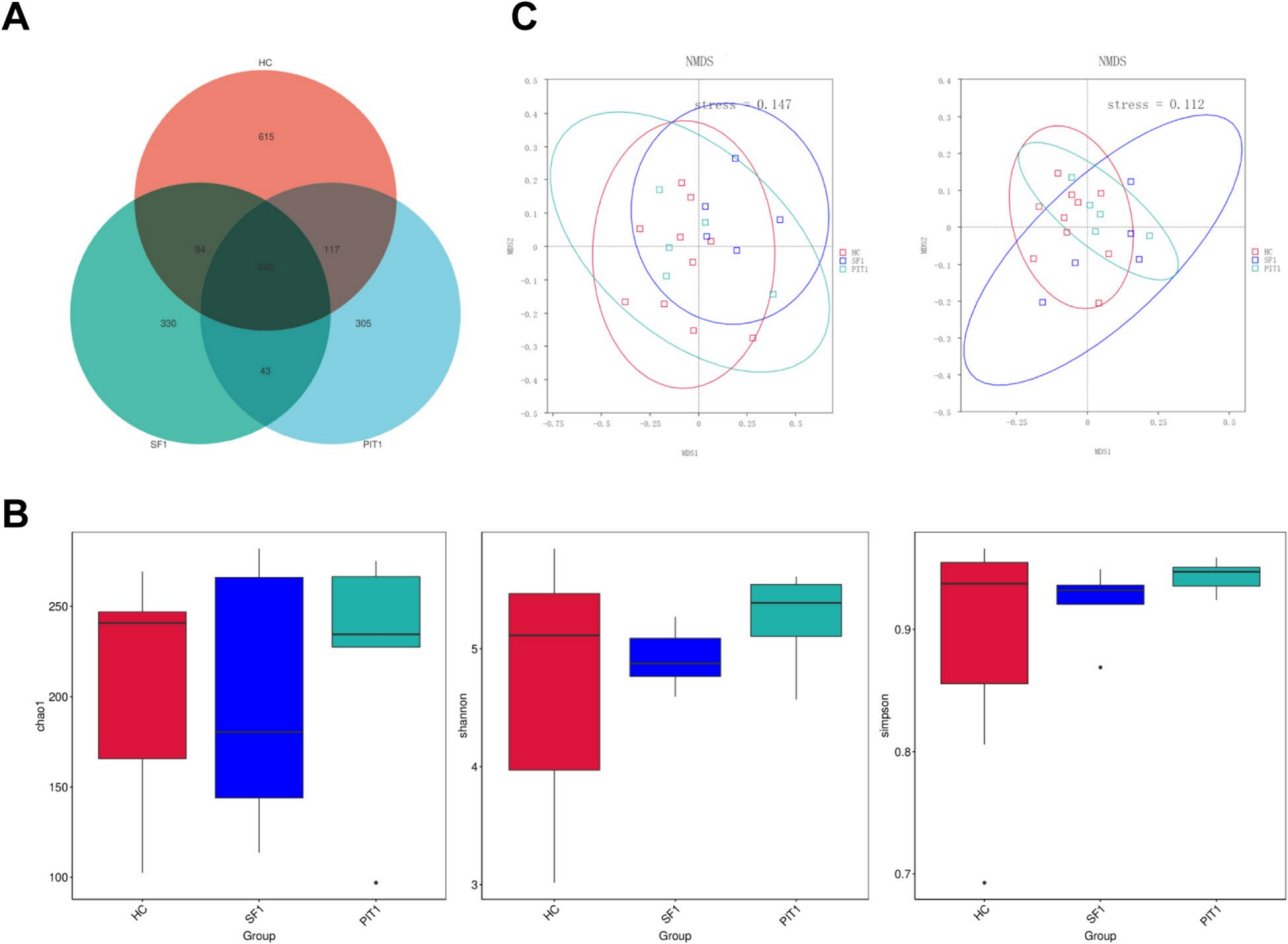
Analysis of gut microbiota diversity. **A** Venn graph of the number of ASVs in each group. **B** The difference of gut microbiota alpha-diversity indices among groups was evaluated by Chao1 index, Shannon index, and Simpson index, *P* > 0.05. **C** The differences in gut microbiota beta diversity among groups were analyzed using NMDS, stress < 0.2

Based on the above research results, we further analyzed whether there are differences in the characteristics of gut microbiota between patients with PIT1, SF-1 lineage PitNETs, and healthy controls. Five patients with PIT1 lineage PitNETs and five patients with SF-1 lineage PitNETs were randomly selected, along with ten corresponding healthy controls. Fecal samples were subjected to 16S rRNA amplicon sequencing to analyze the characteristics of the gut microbiota. The sequencing results showed that the number of ASVs in the HC group was 615, 330 in the SF-1 group, and 305 in the PIT1 group. The number of shared ASVs among the three groups was 240 (Fig. 3A).

### Analysis of gut microbiota diversity

Using the Chao1 index, Shannon index, and Simpson index to evaluate the differences in gut microbiota alpha-diversity indices among the groups, the results showed that there were no significant differences in alpha-diversity indices among the three groups (Fig. 3B). Analysis of the beta-diversity of gut microbiota among the groups using NMDS revealed significant differences in beta-diversity among the three groups (Fig. 3C), suggesting that the composition of the gut microbiota in PitNETs patients differs from that of healthy controls.

### Analysis of gut microbiota characteristics

**Fig. 4:**
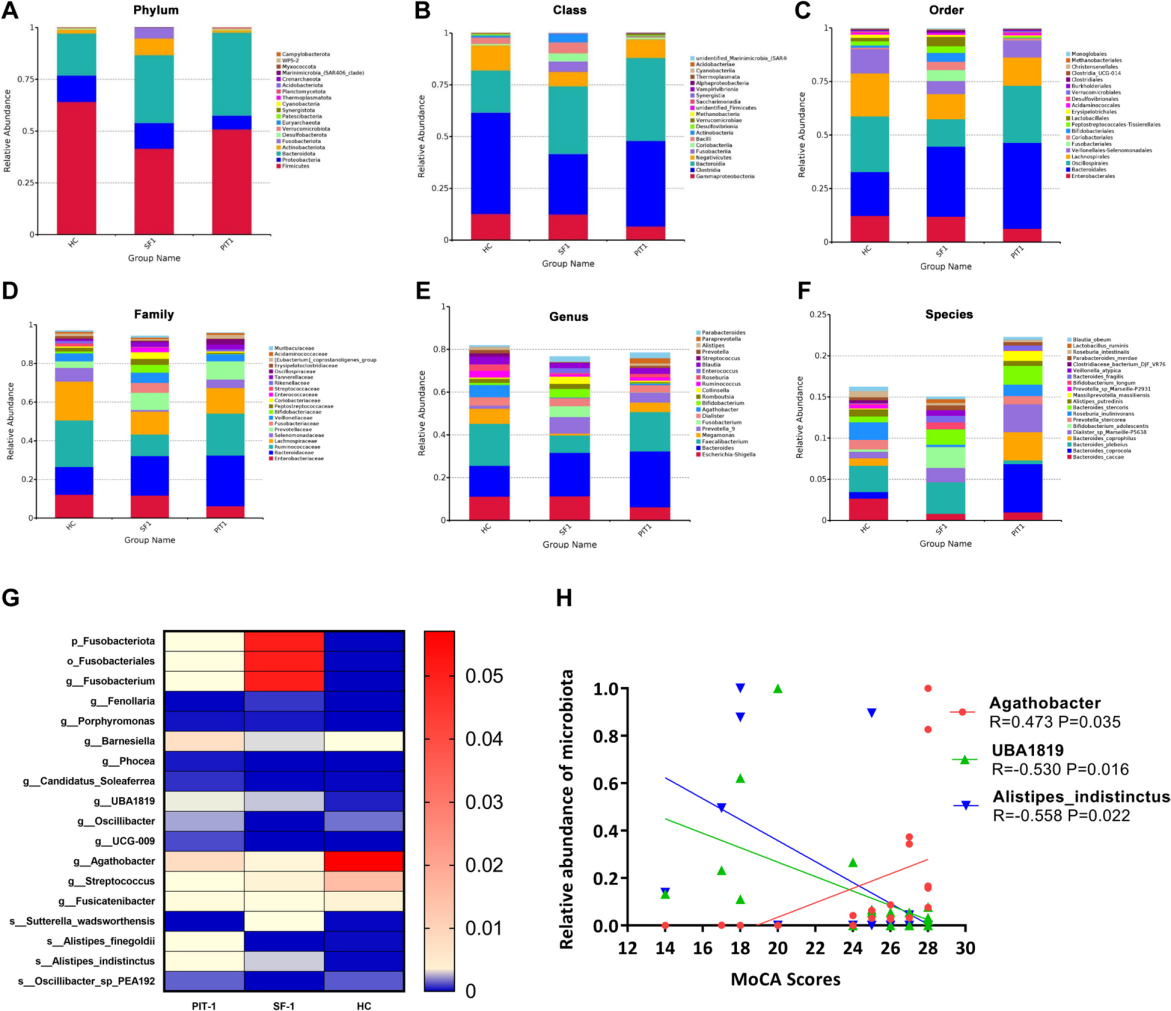
Analysis of gut microbiota characteristics of PitNETs patients by 16S rRNA amplification sequencing. The top 20 most abundant gut microbiota in each group at the phylum (**A**), class (**B**), order (**C**), family (**E**), genus (**D**), and species (**F**) levels. **G** LEfSe analyzed the gut microbiota that contributed the most to inter-group differences at the phyla, order, genus, and species levels. The transition from blue to red indicates a progression from low to high enrichment. **H** Correlation analysis between gut microbiota abundance and cognitive function scores, adjusted *P* < 0.05

The species composition of gut microbiota was analyzed across all groups at multiple taxonomic levels. Figure 4A, B, C, D, E, and F displays the top 20 most abundant microbial taxa at each taxonomic level. LEfSe analysis identified taxa that contributed most significantly to intergroup differences across phylum, class, order, family, genus, and species levels (Fig. 4G).

At the phylum and order levels, Fusobacteriota and Fusobacteriales were significantly enriched in the SF-1 group (adjusted *p*-value < 0.05). At the genus level, *Fusobacterium*, *Pseudocitrobacter*, *Porphyromonas*, and *Fenollaria* were significantly enriched in the SF-1 group, whereas *Barnesiella*, *Phocea*, *Candidatus_soleaferrea*, *UBA1819*, *Oscillibacter*, and *UCG_009* were significantly enriched in the PIT1 group (adjusted *p*-value < 0.05). *Agathobacter* and *Streptococcus* were notably increased in the HC group (adjusted *p*-value < 0.05). At the species levels, *Alistipes_finegoldii*, *Alistipes_indistinctus*, and *Oscillibacter_sp_PEA192* were significantly enriched in the PIT1 group, whereas *Sutterella_wadsworthensis* was notably increased in the SF-1 group (adjusted *p*-value < 0.05).

Based on the analysis of species differences in gut microbiota among the PIT1, SF-1, and HC groups, Spearman correlation analysis was conducted between the differential microbiota at various levels and the total MoCA score. The results indicate that *Agathobacter* is significantly positively correlated with MoCA (*R* = 0.473, adjusted *p*-value = 0.035), while *UBA1819* (*R* = − 0.530, adjusted *p*-value = 0.016) and *Alistipes_indistinctus* (*R* = − 0.558, adjusted *p*-value = 0.022) are significantly negatively correlated with MoCA scores (Fig. 4H).

## Discussion

Neuropsychological deficits are prevalent in patients with PitNETs, with the prevalence of neurocognitive dysfunctions ranging between 15 and 83% in Cushing’s disease [12] and 2%–33% in acromegaly [13], affecting mainly the domains of memory and attention. Memory alterations were observed in 22% of patients with nonfunctional PitNETs [14]. In this study, compared with the healthy control group, patients with PitNETs performed significantly worse in cognitive function, suggesting that patients with PitNETs may have cognitive deficits of varying degrees. If not intervened in time, these deficits may develop into more severe mental disorders. Therefore, the cognitive functioning status of patients with PitNETs should not be ignored, and a comprehensive assessment of the factors affecting cognitive functioning in patients with PitNETs is essential for the prevention and treatment of neuropsychological disorders in patients with PitNETs.

To clarify the causes of CI in patients with PitNETs, we analyzed the relationship between tumor intrinsic characteristics such as tumor volume, invasiveness, hormone levels, and lineage origin and cognitive function. We first analyzed the relationship between tumor volume, invasiveness, and cognitive function in patients with PitNETs. The results revealed that there was no significant association between tumor volume and patients’ cognitive function, which is consistent with previous research findings [14, 15]. Regarding tumor invasiveness, we discovered that there was no significant correlation between the extent of tumor invasion and the cognitive function score of patients. However, Hendrix et al. [16] asserted that tumor expansion in the suprasellar direction was the primary factor contributing to CI in patients with PitNETs. This discrepancy in results may be due to differences in the degree of invasiveness, which requires research and development of a validated scoring system to quantify the degree of tumor invasiveness to evaluate the relationship between invasiveness and cognitive dysfunction.

Hormonal dysregulation may be a critical factor affecting cognitive function in patients with PitNETs. The most frequently observed endocrine abnormalities in these patients involve pathological elevations of GH, PRL, and ACTH. Both GH-secreting and PRL-secreting adenomas are classified as PIT1-lineage PitNETs, whereas ACTH-secreting adenomas belong to the TPIT lineage. Gonadotroph adenomas, which typically do not exhibit significant hormonal hypersecretion, are categorized as SF1-lineage PitNETs. During the patient enrollment period of this study, the number of cases with TPIT-lineage PitNETs was insufficient to meet the minimum sample size required for meaningful statistical analysis, reflecting the relatively low incidence of this subtype compared to PIT1 and SF1 lineages. Consequently, TPIT-lineage patients were excluded from the current analysis to maintain statistical robustness.

Our results demonstrated that cognitive performance was significantly impaired in patients with functional PitNETs relative to those with nonfunctional tumors and more notably reduced in PIT1-lineage PitNETs compared to SF1-lineage tumors. Previous studies have shown that cognitive impairment is highly prevalent in patients with acromegaly [13], and its severity correlates with elevated levels of GH and IGF-1 [17]. Treatment with GH-suppressing analogs has been shown to improve cognitive function in these patients [18]. Importantly, key brain regions involved in cognition (such as the parahippocampal gyrus, amygdala, and prefrontal cortex) express abundant GH and IGF-1 receptors. GH and IGF-1 are known to promote hippocampal neurogenesis, further supporting a close relationship between GH/IGF-1 signaling and cognitive processes [19].

Prolactin has also emerged as a potential modulator of cognitive function. Hyperprolactinemia has been associated with deficits in attention and memory [20]. Genes linked to PRL-induced microglial activation may play a vital role in neuronal protection and hippocampal neuroimmune regulation. Moreover, PRL exerts both pro- and anti-inflammatory effects within the central nervous system, and neuroinflammation is increasingly recognized as a contributor to cognitive decline [21, 22].

Transnasal endoscopic surgery is the most important method for treating PitNETs. In this study, a significant improvement in the MoCA scores was observed in patients with PitNETs following transnasal endoscopic surgery. This improvement may be attributed to the normalization of hormonal levels post-surgery, which alleviates the detrimental effects of abnormally secreted hormones on higher neurological functions. It is important to note that factors such as postoperative hypopituitarism, pharmacological interventions, particularly hormone replacement therapy, and practice effects could potentially influence cognitive function scores. Nevertheless, the endoscopic transnasal surgical treatment can bring benefits for the improvement of cognitive function in patients with PitNETs.

The cognitive status of PitNETs patients may improve after surgery. But some studies have found that some patients’ cognitive function does not improve even after their tumors are effectively treated [23]. So, in addition to inherent characteristics of the tumor itself that may affect cognitive function, what other patient-specific factors might influence cognitive function? Research has shown that gut microbiota is closely related to the development of neuropsychic disorders [23]. The microbiota-gut-brain axis may be involved in CI and dementia by affecting the permeability of the blood–brain barrier (BBB) and inducing neuroinflammation [24]. In germ-free mice receiving fecal transplants from sleep-deprived CI mice, levels of pro-inflammatory factors such as IL-6, IL-1β, and TNF-α significantly increased. Additionally, in diabetic CI mice, beneficial symbiotic bacteria such as *Bifidobacterium* and *Lactobacillus* decreased, while bacteria that may trigger inflammatory responses, such as *Enterococcus* and *Streptococcus*, increased [25]. These studies suggest that dysbiosis can induce systemic inflammation and BBB damage by upregulating pro-inflammatory factors. Pro-inflammatory factors can cross the damaged BBB, exacerbate neuroinflammation, and ultimately disrupt the normal function of neural networks, negatively impacting cognitive function.

Through 16S rRNA sequencing analysis of fecal samples from PitNETs patients and healthy controls, we found that the gut microbiome structure of PitNETs patients differed from that of the control group. Specifically, there was no significant difference in the richness and evenness of the gut microbiome between PitNETs patients and the control group, while there were differences in the structure and quantity of the gut microbiome among the three groups, indicating that the composition of the gut microbiome had changed. The generation of such differences may be related to a variety of factors such as diet, age, gender, hormone levels, and disease status [26]. A study by Jensen et al. noted that changes in GH can lead to significant differences in the composition of the gut microbiome in mice. The gut microbiome, in turn, can affect the host’s hormone levels, such as growth hormone-releasing peptide and cortisol, indicating a complex interaction between the GH/IGF-1 axis and the gut microbiome [27]. Therefore, hormonal imbalances in PitNETs patients may lead to dysregulation of the gut microbiome, which can modulate mood, cognition, and pain perception.

The LEfSe analysis results showed that *Agathobacter* was significantly enriched in the HC group and positively correlated with MoCA scores, while *UBA1819* and *Alistipes indistinctus* were significantly enriched in the PIT1 group and negatively correlated with MoCA scores. *Agathobacter* is an anaerobic gram-positive bacterium belonging to the family Lachnospiraceae. Studies have found that the content of *Agathobacter* in the gut microbiota of female patients with depression [28] and cerebral ischemic stroke [29] is significantly reduced. In children with moderate acute malnutrition, the abundance of *Agathobacter* in their feces significantly increased after receiving microbiota-directed complementary food, and their developmental delays, including cognitive deficits, significantly improved [30]. The products of *Agathobacter* include butyrate, acetate, hydrogen, and lactate. Butyrate, as a short-chain fatty acid, not only provides energy to the body but also regulates the permeability of the BBB and maintains the homeostasis of the central nervous system [31]. In the healthy control group, *Agathobacter* is significantly enriched, which helps maintain normal cognitive function.

### *UBA1819* belongs to the genus within the family Ruminococcaceae

Studies have shown that the abundance of Ruminococcaceae increases in mice with behavioral deficits and is closely associated with anxiety-like behavior [32]. *Alistipes indistinctus* belongs to the genus *Alistipes*, and the levels of *Alistipes* are moderately to strongly correlated with the production of IL-1, IL-6, and TNF-α and are involved in promoting inflammation and tumorigenesis [33]. The abundance of *Alistipes indistinctus* has been linked to the long-term development of depression [34]. *UBA1819* and *Alistipes indistinctus* are enriched in the PIT1 group, which may lead to impaired gut barrier function and the production of abnormal metabolic products, exacerbating cognitive dysfunction.

Based on the exploratory findings of this study, the observed increase in potentially pro-inflammatory taxa and decrease in beneficial bacteria in patients with PitNETs suggest a possible link to cognitive dysfunction. It is hypothesized that gut microbiota alterations may influence cognitive function through a proposed gut–brain axis mechanism, whereby microbial metabolites could stimulate pro-inflammatory cytokine release, increase intestinal barrier permeability, compromise blood–brain barrier integrity, and potentially contribute to neuroinflammation. These preliminary observations warrant further validation in future large-scale studies.

## Conclusions

**Fig. 5:**
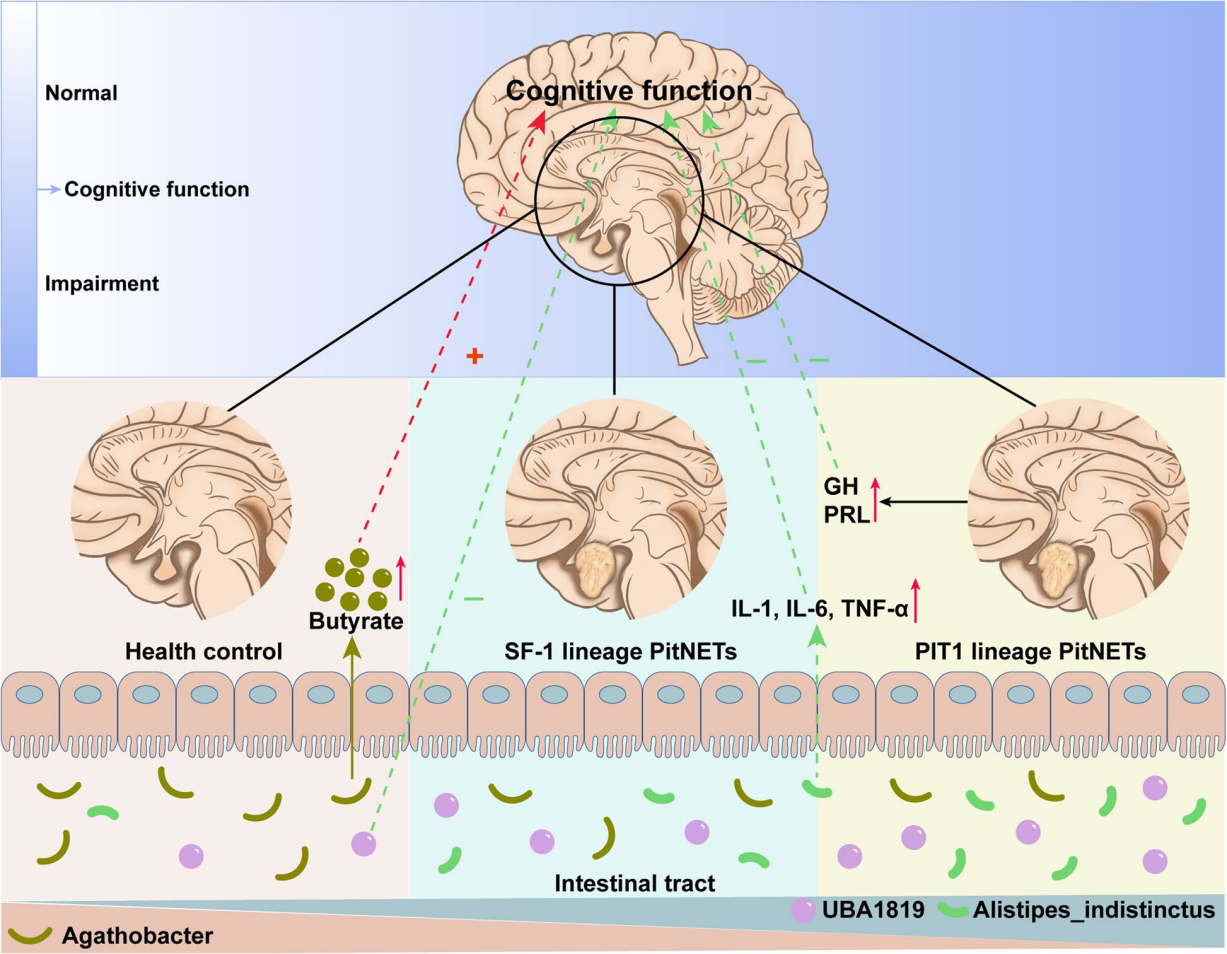
Hypothetical model of the potential interactions among hormone levels, gut microbiota, and cognitive function in patients with PitNETs. The red plus sign denotes a positive association with cognitive function; the green minus sign denotes a negative association with cognitive function

Patients with PitNETs may exhibit varying degrees of cognitive impairment, which is more prevalent among those with PIT1 lineage PitNETs. This phenomenon may be associated with hormone dysregulation induced by these tumors, as well as alterations in the gut microbiome composition, such as reduced abundance of the butyrate-producing genus *Agathobacter* and increased levels of *UBA1819* and *Alistipes indistinctus*, taxa that have been linked to inflammatory states. To improve cognitive function in PitNETs patients, in addition to active tumor management (primarily surgical intervention), future research should *also explore the potential role of gut microbiota characteristics* (Fig. 5).

### Limitations

This study has several limitations that should be considered when interpreting the findings. The sample size, particularly in the gut microbiome cohort, is modest and may limit the generalizability and statistical power of the results. PERMANOVA analysis confirmed substantial household clustering (*R*^2^ = 0.504), though this effect did not reach statistical significance (*q* = 0.13), potentially reflecting power limitations in the permutation-based test with 10 families. Although household-matched controls were implemented to reduce environmental confounders, residual clustering effects may persist. The cross-sectional design and absence of longitudinal microbiome data preclude causal inferences regarding microbial dynamics and cognitive outcomes. Additionally, while the MoCA is appropriate for cognitive screening, it may not capture subtle domain-specific deficits. Although comprehensive contaminant removal was not feasible, the biological consistency of our findings and their alignment with established gut-brain axis mechanisms support their potential relevance. These limitations highlight the preliminary nature of this investigation and underscore the need for larger, longitudinal studies to validate and extend these findings.

## Supplementary Information

Supplementary Material 1: Table 1 Results of differential abundance analysis of gut microbiota across study groups.

Supplementary Material 2: Table 2 Pre-operative and post-operative pituitary hormone levels in patients with PitNETs.

Supplementary Material 3: Ouput.csv.

Supplementary Materials 4: otu.pairwise.adonis.

## Data Availability

The raw sequencing data generated in this study have been deposited in NCBI Sequence Read Archive (http://www.ncbi.nim.nih.gov/sra) under the accession numbers PRJNA1169806.

## Abbreviations

PitNETs: Pituitary neuroendocrine tumors
CI: Cognitive impairment
MoCA: Montreal Cognitive Assessment
MCI: Mild cognitive impairment
GH: Growth hormone
IGF-1: Insulin-like growth factor-1
FSH: Follicle-stimulating hormone
LH: Luteinizing hormone
PRL: Prolactin
TSH: Thyroid-stimulating hormone
F-PitNETs: Functional PitNETs
NF-PitNETs: Nonfunctional PitNETs
PPIs: Proton-pump inhibitors
I-PitNETs: Invasive PitNETs
NI-PitNETs: Noninvasive PitNETs
NMDS: Nonmetric multidimensional scaling
LEfSe: LDA effect size
FDR: False discovery rate
