# Supplementary material for: Distinct gut microbial profile in PIT1 lineage PitNETs: a potential link to cognitive impairment
Source: Chin Neurosurg J. 2025 Dec 30;11:33. doi: 10.1186/s41016-025-00421-7 (PMC12750663; doi:10.1186/s41016-025-00421-7)
Supplement: Supplementary file 1 — Supplementary Material 1: Table 1 Results of differential abundance analysis of gut microbiota across study groups. [file 41016_2025_421_MOESM1_ESM.doc]

****Supplementary Table 1 Results of differential abundance analysis of gut microbiota across study groups.****

| **Taxonomy** | **PIT-1**  **(n=5)** | **SF-1**  **(n=5)** | **HC (n=10)** | **PIT-1 VS SF-1** | | **PIT-1 VS HC** | | **SF-1 VS HC** | |
| --- | --- | --- | --- | --- | --- | --- | --- | --- | --- |
|  |  |  |  | **Raw P Value** | **Adjusted P Value** | **Raw P Value** | **Adjusted P Value** | **Raw P Value** | **Adjusted P Value** |
| p_Fusobacteriota | 0.0004057 | 0.05043 | 3.1e-006 | 0.0604 | 0.0905 | 0.5812 | 0.5812 | 0.0065 | **0.0196** |
| c_Fusobacteriia | 6.193e-005 | 0 | 0.02798 | 0.4676 | 0.6117 | 0.1782 | 0.5345 | 0.6117 | 0.6117 |
| o_Fusobacteriales | 0.0004057 | 0.05043 | 3.1e-006 | 0.0604 | 0.0905 | 0.5812 | 0.5812 | 0.0065 | **0.0196** |
| f__Fusobacteriaceae | 0 | 6.193e-005 | 0.02798 | 0.439 | 0.6585 | 0.2972 | 0.6585 | 0.8816 | 0.8816 |
| g__Fusobacterium | 0.0004057 | 0.05043 | 0 | 0.0492 | 0.0738 | 0.3722 | 0.3722 | 0.0016 | **0.0047** |
| g__Fenollaria | 3.1e-006 | 8.361e-005 | 0 | 0.0786 | 0.118 | 0.453 | 0.453 | 0.0054 | **0.0163** |
| g__Pseudocitrobacter | 0 | 6.812e-005 | 0 | 0.0402 | 0.0603 | >0.9999 | >0.9999 | 0.0178 | 0.0535 |
| g__Porphyromonas | 2.787e-005 | 3.716e-005 | 0 | 0.527 | 0.527 | 0.0961 | 0.1442 | 0.0166 | **0.0499** |
| g__Barnesiella | 0.007073 | 0.0003437 | 0.0004227 | 0.0802 | 0.1203 | 0.0134 | **0.0402** | 0.6507 | 0.6507 |
| g__Papillibacter | 2.476e-005 | 0 | 0 | 0.0399 | 0.0598 | 0.0177 | 0.053 | >0.9999 | >0.9999 |
| g__Phocea | 3.714e-005 | 0 | 0 | 0.0099 | **0.0148** | 0.0029 | **0.0087** | >0.9999 | >0.9999 |
| g__Candidatus_Soleaferrea | 7.431e-005 | 3.1e-006 | 9.29e-006 | 0.0281 | **0.0421** | 0.0185 | **0.0421** | 0.8563 | 0.8563 |
| g__UBA1819 | 0.0003716 | 0.0003097 | 4.799e-005 | 0.057 | 0.0855 | 0.0043 | **0.0128** | 0.5097 | 0.5097 |
| g__Oscillibacter | 0.0002601 | 0 | 0.0001765 | 0.0144 | **0.0433** | 0.0888 | 0.1332 | 0.2616 | 0.2616 |
| g__UCG-009 | 0.0001146 | 0 | 0 | 0.0099 | **0.0148** | 0.0029 | **0.0087** | >0.9999 | >0.9999 |
| g__Agathobacter | 0.007893 | 0.00235 | 0.05709 | 0.258 | 0.258 | 0.1812 | 0.258 | 0.0082 | **0.0246** |
| g__Streptococcus | 0.0004955 | 0.002936 | 0.01489 | 0.1814 | 0.2722 | 0.0124 | **0.0373** | 0.3387 | 0.3387 |
| g__Fusicatenibacter | 0.0009104 | 0.0008701 | 0.003055 | 0.9147 | 0.9147 | 0.0304 | **0.0457** | 0.0221 | **0.0457** |
| s__Sutterella_wadsworthensis | 0 | 0.0004738 | 9.29e-006 | 0.0197 | **0.0345** | 0.6749 | 0.6749 | 0.023 | **0.0345** |
| s__Clostridium_paraputrificum | 0 | 8.36e-005 | 0 | 0.0402 | 0.0603 | >0.9999 | >0.9999 | 0.0178 | 0.0535 |
| s__Ruminococcaceae_bacterium_GD6 | 2.478e-005 | 0 | 0 | 0.0402 | 0.0603 | 0.0178 | 0.0535 | >0.9999 | >0.9999 |
| s__Prevotella_sp_AG487_50_53 | 0.0008516 | 0 | 0 | 0.0402 | 0.0603 | 0.0178 | 0.0535 | >0.9999 | >0.9999 |
| s__Alistipes_finegoldii | 0.0006038 | 0 | 1.393e-005 | 0.016 | **0.0242** | 0.0161 | **0.0242** | 0.7075 | 0.7075 |
| s__Alistipes_indistinctus | 0.0008949 | 0.0003189 | 7.74e-006 | 0.0519 | 0.0778 | 0.0046 | **0.0138** | 0.5554 | 0.5554 |
| s__Oscillibacter_sp_PEA192 | 0.0001517 | 0 | 0.0001394 | 0.0121 | **0.0362** | 0.0701 | 0.1051 | 0.2771 | 0.2771 |
