# Supplementary material for: Distinct gut microbial profile in PIT1 lineage PitNETs: a potential link to cognitive impairment
Source: Chin Neurosurg J. 2025 Dec 30;11:33. doi: 10.1186/s41016-025-00421-7 (PMC12750663; doi:10.1186/s41016-025-00421-7)
Supplement: Supplementary file 2 — Supplementary Material 2: Table 2 Pre-operative and post-operative pituitary hormone levels in patients with PitNETs. [file 41016_2025_421_MOESM2_ESM.doc]

****Supplementary Table 2**** Pre-operative and post-operative pituitary hormone levels in patients with PitNETs.

| Number | **Preoperation** | | | | | | | |  | **Three months after the operation** | | | | | | | |
| --- | --- | --- | --- | --- | --- | --- | --- | --- | --- | --- | --- | --- | --- | --- | --- | --- | --- |
|  | GH (ng/ml) | IGF-1 (ng/ml) | PRL(ng/ml) | FSH (mIU/ml) | LH (mIU/ml) | TSH (mIU/L) | 8am Cortisol (pg/mL) | 8am ACTH (ng/ml) |  | GH (ng/ml) | IGF-1 (ng/ml) | PRL(ng/ml) | FSH (mIU/ml) | LH (mIU/ml) | TSH (mIU/L) | 8am Cortisol (pg/mL) | 8am ACTH (ng/ml) |
| NO.1 | 6.68 | 485 | 14.4 | 7.19 | 1.28 | 0.78 | 186 | 10.5 |  | 2.37 | 289 | 12.9 | 8.81 | 1.65 | 0.84 | 189.6 | 13.9 |
| NO.2 | 12.8 | 613 | 89.3 | 8.77 | 1.61 | 2.27 | 185.6 | 26.92 |  | 0.1 | 187 | 2.4 | 6.55 | 1.34 | 1.57 | 135.5 | 18.67 |
| NO.3 | 43.1 | 642 | 99.9 | 3.02 | 1.1 | 1.04 | 224.6 | 38.96 |  | 4.46 | 286 | 16 | 6.68 | 2.05 | 1.04 | 93.51 | 13.4 |
| NO.4 | 2.16 | 428 | 4 | 8.61 | 7.14 | 0.07 | 11.76 | 0.54 |  | 2.37 | 310 | 14.9 | 6.02 | 4.68 | 0.89 | 200 | 24 |
| NO.5 | 13.8 | 628 | 7.3 | 4.79 | 4.25 | 1.05 | 147.5 | 23.99 |  | 0.49 | 157 | 7 | 3.76 | 2.45 | 0.55 | 9.94 | 10.34 |
| NO.6 | 13.7 | 617 | 10 | 68.56 | 20.57 | 0.88 | 114 | 10.24 |  | 0.43 | 218 | 13.4 | 34.76 | 16.97 | 0.49 | 164.69 | 9.45 |
| NO.7 | 20.4 | 638 | 54.9 | 3.03 | 1.45 | 0.62 | 179.6 | 15.63 |  | 1.36 | 252 | 10.96 | 5.6 | 0.9 | 1.08 | 152 | 21.6 |
| NO.8 | 6.55 | 534 | 53.7 | 7.54 | 2.44 | 1.8 | 151 | 18.79 |  | 0.4 | 196 | 20.4 | 5.78 | 1.38 | 1.82 | 177.5 | 13.35 |
| NO.9 | 3.03 | 496 | 7.3 | 4.2 | 2.45 | 1.13 | 150.6 | 30.8 |  | 0.23 | 159 | 4.39 | 3.5 | 2.3 | 4.93 | 128 | 23.7 |
| NO.10 | 5.35 | 447 | 9.1 | 5.96 | 4.65 | 1.94 | 129.3 | 21.25 |  | 3.79 | 350 | 4.6 | 4.48 | 3.7 | 0.75 | 64.2 | 11.1 |
| NO.11 | 4.32 | 510 | 21 | 4.22 | 1.08 | 1.72 | 185.3 | 8.45 |  | 3.4 | 412 | 16.4 | 10.14 | 7.94 | 0.57 | 155 | 5.67 |
| NO.12 | 4.7 | 496 | 129.4 | 4.84 | 2.91 | 4.92 | 163.3 | 15.48 |  | 4.8 | 589 | 24.5 | 1.31 | 1.62 | 0.5 | 159.5 | 4.87 |
| NO.13 | 69.5 | 681 | 79.3 | 1.94 | 0.38 | 0.01 | 116 | 7.35 |  | 0.8 | 215 | 31.1 | 4 | 0.58 | 0.02 | 170.5 | 10.08 |
| NO.14 | 46.5 | 598 | 13.5 | 43.81 | 13.47 | 0.93 | 158.4 | 25.23 |  | 3.3 | 403 | 8.88 | 24.3 | 6.6 | 1.19 | 218.7 | 31.66 |
| NO.15 | 18.7 | 622 | 11.7 | 8.28 | 3.2 | 2 | 154.6 | 25.98 |  | 1.05 | 331 | 3.7 | 8.85 | 3.59 | 1.67 | 102.4 | 4.2 |
| NO.16 | 0.12 |  | 173.1 | 3.32 | 0.96 | 0.94 | 137 | 9.56 |  | 0.41 |  | 4.5 | 5.46 | 4.52 | 2.04 | 213.9 | 13.05 |
| NO.17 | 0.27 |  | 485 | 7.43 | 4.33 | 5.98 | 227.9 | 17.62 |  | 0.77 |  | 99.5 | 8.69 | 5.78 | 0.31 | 179.4 | 1.47 |
| NO.18 | 0.11 |  | 803 | 3.58 | 2.98 | 2.92 | 153.6 | 20.56 |  | 0.53 |  | 241 | 5.44 | 6.07 | 2.31 | 211.8 | 30 |
| NO.19 | 0.61 |  | 990 | 1.9 | 0.53 | 1.89 | 19.39 | 11.99 |  | 0.61 |  | 187 | 1.9 | 0.53 | 0.58 | 20.52 | 2.92 |
| NO.20 | 0.05 |  | 376 | 3.48 | 2.01 | 2.01 | 153.4 | 19.71 |  | 0.32 |  | 1.3 | 3.4 | 3.08 | 1.83 | 155.5 | 38.76 |
| NO.21 | 0.07 |  | 165.5 | 4.42 | 1.73 | 3.61 | 224 | 22.27 |  | 0.19 |  | 6.7 | 5.82 | 4.69 | 2.03 | 188.1 | 10.78 |
| NO.22 | 1.01 |  | 398 | 3.1 | 1.5 | 0.64 | 204 | 22.12 |  | 0.05 |  | 155.2 | 2.58 | 1.53 | 1.85 | 215.3 | 33.73 |
| NO.23 | 0.24 |  | 38.2 | 1.41 | 0.27 | 0.93 | 32.03 | 3.06 |  | 0.07 |  | 25.2 | 0.59 | 0.2 | 0.14 | 0.98 | 3.79 |
| NO.24 | 0.13 |  | 762 | 2.94 | 0.38 | 2.31 | 136.5 | 9.85 |  | 0.39 |  | 376 | 4.43 | 1.15 | 3.69 | 21.94 | 7.42 |
| NO.25 | 0.43 |  | 10.7 | 4.52 | 2.23 | 0.74 | 90.15 | 12.75 |  | 0.3 |  | 11.8 | 4.08 | 2.34 | 0.86 | 93.65 | 11.88 |
| NO.26 | 0.05 |  | 23.4 | 80.89 | 3.14 | 8.32 | 172.7 | 24.57 |  | 0.08 |  | 7.8 | 4.75 | 4.47 | 5.84 | 135.1 | 22.86 |
| NO.27 | 0.55 |  | 24.5 | 6.07 | 3.37 | 2.57 | 149.1 | 15.6 |  | 0.45 |  | 15.4 | 4.59 | 1.59 | 0.87 | 25.48 | 2.4 |
| NO.28 | 0.06 |  | 24.5 | 11.32 | 2.02 | 4.14 | 113.5 | 6.12 |  | 0.56 |  | 26.7 | 6.62 | 2.25 | 3.03 | 264.9 | 22.72 |
| NO.29 | 0.11 |  | 29.4 | 6.18 | 3.08 | 5.42 | 159.4 | 16.15 |  | 0.36 |  | 3.2 | 6.7 | 3.24 | 1.97 | 144 | 24 |
| NO.30 | 0.05 |  | 25.5 | 4.47 | 1.28 | 2.99 | 36.06 | 4.42 |  | 0.05 |  | 8.9 | 6.14 | 1.49 | 2.98 | 127.8 | 18.2 |
| NO.31 | 0.57 |  | 15.2 | 39.16 | 10.89 | 7.95 | 116.4 | 10.21 |  | 0.87 |  | 5.5 | 31.71 | 8.47 | 4.24 | 294.1 | 15.44 |
| NO.32 | 0.05 |  | 16.1 | 4.16 | 1.59 | 1.77 | 27.23 | 6.57 |  | 0.24 |  | 8.2 | 4.01 | 2.5 | 0.79 | 169.7 | 12.53 |
| NO.33 | 0.05 |  | 3.5 | 6.25 | 0.95 | 1.24 | 177.5 | 16.64 |  | 0.05 |  | 8.2 | 1.9 | 1.91 | 1.42 | 86.56 | 17.5 |
| NO.34 | 0.38 |  | 7.5 | 5.2 | 0.82 | 0.26 | 156.3 | 33.03 |  | 0.08 |  | 7.7 | 4.68 | 1.22 | 2.12 | 155.2 | 20.44 |
| NO.35 | 0.15 |  | 21.3 | 5.53 | 3.52 | 2.73 | 82.85 | 12.22 |  | 0.47 |  | 8.2 | 3.4 | 3.93 | 1.96 | 146 | 17 |
| NO.36 | 0.43 |  | 16.6 | 11.82 | 3.79 | 1.91 | 121.2 | 4.2 |  | 0.92 |  | 6.8 | 9.51 | 3.54 | 0.48 | 271.2 | 10.21 |
| NO.37 | 0.1 |  | 26 | 22.54 | 6.19 | 0.2 | 223.4 | 12.98 |  | 0.35 |  | 18.4 | 12.61 | 4.25 | 0.27 | 114.7 | 16.54 |
| NO.38 | 0.07 |  | 2.1 | 3.21 | 0.97 | 0.03 | 27.56 | 7.56 |  | 0.05 |  | 2.4 | 3.83 | 1.72 | 3.39 | 110 | 19.46 |
| NO.39 | 0.05 |  | 21.7 | 4.43 | 1 | 5.45 | 70.69 | 9.69 |  | 0.1 |  | 7 | 1.53 | 0.59 | 4.32 | 9.47 | 3.38 |
| NO.40 | 0.09 |  | 13.9 | 2.24 | 0.52 | 6.03 | 39.89 | 10.83 |  | 0.13 |  | 10 | 3.16 | 1.1 | 2.64 | 141.4 | 28.33 |
| NO.41 | 0.05 |  | 23.2 | 3.44 | 1.22 | 5.49 | 92.92 | 14.41 |  | 0.05 |  | 11.4 | 3.06 | 1.06 | 2.81 | 25.27 | 3.2 |
| NO.42 | 0.26 |  | 23.4 | 19.47 | 1.88 | 4.18 | 127.3 | 16.46 |  | 0.15 |  | 11.3 | 4.51 | 3.04 | 3.39 | 123.6 | 13.28 |
